# Progress in Bi_2_WO_6_-Based Materials for Electrochemical Sensing and Supercapacitor Applications

**DOI:** 10.3390/molecules30153149

**Published:** 2025-07-28

**Authors:** Khursheed Ahmad, Dhanabalan Karmegam, Tae Hwan Oh

**Affiliations:** School of Chemical Engineering, Yeungnam University, Gyeongsan 38541, Republic of Korea; dhanabalan_nano@yu.ac.kr

**Keywords:** Bi_2_WO_6_, energy storage, electrochemical sensors, supercapacitors

## Abstract

Recently, the design and fabrication of novel electrode materials for electrochemical and electronic devices have received the widespread attention of the scientific community. In particular, electrochemical sensors and supercapacitors (SCs) involve the use of catalysts, which can enhance the electrochemical reactions at the surface of the electrode. Bismuth tungstate (Bi_2_WO_6_) is a cost-effective and efficient electrode material with decent optoelectronic properties and stability. The properties of Bi_2_WO_6_ can be improved by incorporating carbon-based materials, and the resulting composite may be a promising electrode material for electrochemical sensing and SCs. As per the available reports, Bi_2_WO_6_ has been combined with various nanostructured and conductive materials for electrochemical sensing and SC applications. This review discusses synthetic methods for the preparation of Bi_2_WO_6_. Progress in the construction of hybrid composites for electrochemical sensing and SC applications is reviewed. The Conclusion section discusses the role of electrode materials and their limitations with future perspectives for electrochemical sensing and SCs. It is believed that the present review may be useful for researchers working on Bi_2_WO_6_-based materials for electrochemical sensing and SC applications.

## 1. Introduction

Previously, nanostructured materials have received enormous interest for a variety of optoelectronic applications, such as electrochemical sensors [1], photocatalytic [2], optical [3], energy storage [4] and photovoltaics [5]. Nanostructured materials possess decent conductive properties, electrocatalytic properties, stability, and efficient specific surface area, which makes them desirable materials for such optoelectronic applications [6,7,8]. Semiconducting metal oxides are stable electrode materials for various electrochemical applications such as energy storage [9,10] and sensors/biosensors [11,12]. In particular, bismuth tungstate (Bi_2_WO_6_) is a semiconducting metal oxide and exhibits excellent physicochemical properties, such as photoluminescence [13,14], photocatalysis [15,16], pyroelectric [17], nonlinear dielectric induction [18], piezoelectric [19], and ferroelectric [20]. In previous years, various methods were utilized for the formation of Bi_2_WO_6_, including the solvothermal method [21,22], co-precipitation method [23], solid phase preparation method [24], ion exchange method [25], sol–gel method [26], ultrasonic spray pyrolysis method [27], hydrothermal method [28], ultrasonic chemical method [29], electro-spinning method [30], and microwave method [31]. It is well known that each method has its own limitations, disadvantages, and advantages. Bi_2_WO_6_ formed via different methods has different surface morphologies and particle sizes [32]. Thus, it is worth mentioning that the properties of Bi_2_WO_6_ can be tuned by employing a suitable synthetic method. In previous years, Bi_2_WO_6_ and its composites were explored in catalysis [33], energy conversion [34], gas sensors [35], dye degradation [36], hydrogen production [37], oxygen evolution reactions [38], CO oxidation [39], adsorption [40], and biomedical [41] applications. It is believed that Bi_2_WO_6_-based materials may be efficient electrode materials for the development of electrochemical sensors and energy storage devices.

At present, energy consumption has been significantly enhanced due to growth in the global population and the modernization of society [42]. Fossil fuels are extensively utilized to supply energy but have some limitations, such as limited abundance and the contribution of fossil fuels to environmental pollution [43]. Therefore, renewable energy-based technologies should be developed by exploring innovative approaches and strategies. In this regard, various energy technologies were developed, with supercapacitors (SCs) bridging the gap between batteries and traditional capacitors [44]. SCs have the potential to store and release energy within a short time, making them a desirable candidate for energy storage applications [45]. Numerous materials, such as transition metal oxides, polymers and semiconducting materials, are used for electrochemical applications [1,4]. It was observed that the utilization of new electrode materials for the fabrication of electrochemical devices is of particular importance. In this regard, Bi_2_WO_6_ emerged as a promising electrode material for sensing and SC applications. It is understood that Bi_2_WO_6_ offers several advantages, including environmental friendliness, decent stability, redox properties, and electrocatalytic properties. The incorporation of Bi_2_WO_6_ with other conductive materials also exhibits improved synergistic interactions and conductive properties, with more electrochemically active sites for redox reactions. Thus, it can be concluded that Bi_2_WO_6_ is a promising electrode material for electrochemical applications. Bi_2_WO_6_-based materials with semiconducting metal oxides, MXenes, LDH, rGO, etc., were utilized for electrochemical sensors and SC applications.

**Chart 1:**
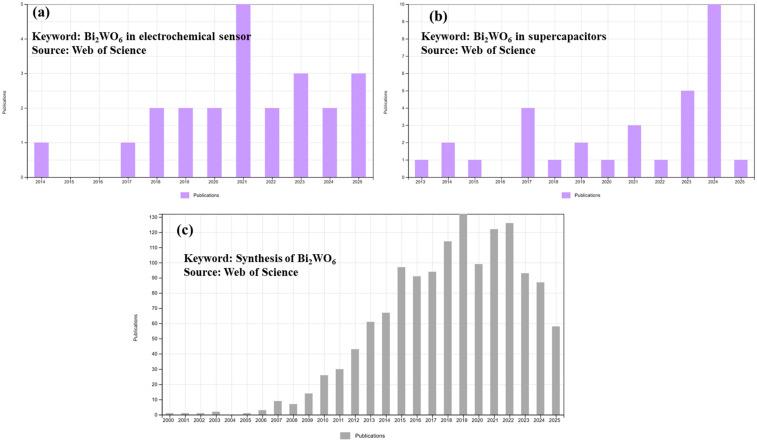
Bar graph shows reports on Bi_2_WO_6_-based (**a**) electrochemical sensors and (**b**) SCs. (**c**) Bar graph shows reports on the synthesis of Bi_2_WO_6_. Source: Web of Science.

In this review, we report the progress in the design and construction of Bi_2_WO_6_-based materials with MXenes, semiconducting metal oxides, LDH, carbon nanotubes, rGO, etc., for sensing and SC applications. The examined literature on Bi_2_WO_6_-based materials for electrochemical sensing and SCs is shown in Chart 1a and Chart 1b, respectively. The reported literature on the synthesis of Bi_2_WO_6_ is summarized in Chart 1c. It can be observed that numerous reports are available on the synthesis of Bi_2_WO_6_, but limited reports are available on the fabrication of Bi_2_WO_6_-based electrochemical sensors and SCs.

## 2. Synthesis Methods

In the last few years, various methods, including electrochemical, hydrothermal, solvothermal, microwave, and sol–gel, were utilized for the preparation of Bi_2_WO_6_ and its composites. In this section, we briefly discuss the synthesis of Bi_2_WO_6_-based materials using different methods.

### 2.1. Hydrothermal Method

The hydrothermal method needs an autoclave/sealed container to improve the reaction between tungsten and bismuth precursors using water as a solvent. In a previous report, flower-like Bi_2_WO_6_ was synthesized through the hydrothermal method [46]. The bismuth nitrate and sodium tungstate precursors were dissolved in Milli-Q water and transferred to the autoclave system. The autoclave reactor was tightly sealed and kept at 160 °C for 12 h in a furnace, which yielded flower-like Bi_2_WO_6_. In contrast, the authors also observed that hydrothermal treatment at 200 °C for 24 h yielded Bi_2_WO_6_ NPs. It is clear that the morphology of Bi_2_WO_6_ can be tuned by optimizing the temperature, pH and reaction time. In another study [47], nickel (Ni)-doped Bi_2_WO_6_ was prepared through the hydrothermal method, possessing a hydrangea-like surface structure. The hydrothermal treatment at 160 °C for 4 h yielded Ni-doped Bi_2_WO_6_. The effects of pH were also studied by Wang et al. [48] to obtain Bi_2_WO_6_ NPs. However, this study used a relatively higher temperature of 200 °C for 20 h. It is noted that the hydrothermal method may be considered ecofriendly if the reaction is carried out at less than 100 °C with reduced time. But the abovementioned study involved 200 °C for 20 h, which is the major limitation for practical applications and scalability. Li et al. [49] reported the synthesis of Bi_2_WO_6_ using the hydrothermal method, which involves a relatively low temperature of 150 °C (time = 6 h). Gao et al. [50] utilized the hydrothermal method for the preparation of copper (Cu)-doped Bi_2_WO_6_. The authors also optimized the pH of the reaction solution, and hydrothermally grown Cu-doped Bi_2_WO_6_ exhibited a flower-like surface morphology under optimized conditions. Metal precursors are dissolved in water, and the pressure in the hydrothermal reactor facilitates the dissolution of precursors in solvents and promotes the nucleation process of the preparation of Bi_2_WO_6_. The hydrothermal method has various advantages, such as tuning the reaction time, pH, concentration of precursors, and temperature, and the high pressure in the autoclave has a significant role in optimizing the surface morphology of Bi_2_WO_6_.

### 2.2. Solvothermal Method

The solvothermal method is a similar approach for the preparation of nanostructured materials, except for the use of different solvents. In hydrothermal methods, water is utilized as a solvent, whereas the solvothermal method involves the use of solvents such as ethylene glycol. In a published research article, the solvothermal method was explored for the fabrication of a titanium dioxide (TiO_2_) nanotube (NT)/Bi_2_WO_6_ composite [51]. Boron (B)-doped Bi_2_WO_6_ was obtained through the solvothermal method by utilizing ethylene glycol (EG) as a solvent [52]. The temperature for the solvothermal treatment was fixed at 433 K for 12 h. In another study [53], it was found that the solvothermal treatment of bismuth nitrate pentahydrate and sodium tungstate dehydrate in the presence of an EG solvent at 160 °C for 24 h yielded a hollow-shaped Bi_2_WO_6_ structure. Wu et al. [54] also explored novel strategies such as the microwave-assisted solvothermal approach towards the fabrication of Bi_2_WO_6_ nanosheets. The working principle for the solvothermal method is similar to the hydrothermal method. Thus, it is clear that the solvothermal method also offers several advantages, such as the optimization of pH, temperature, precursor concentration, and reaction time, to tune the surface morphology of the nanostructured materials.

### 2.3. Electrochemical Method

Electrodeposition, which is also called the electrochemical method, has been explored for the preparation of nanostructured materials and polymers. Zhu et al. [55] utilized the electrochemical method for the preparation of a Bi_2_WO_6_/rGO composite using a three-electrode assembly. The authors used a −1.2 V potential for the electrodeposition process. Indium tin oxide (ITO) was used as a working electrode substrate, whereas platinum foil was adopted as a counter electrode, with silver/silver chloride (Ag/AgCl) as a reference electrode. However, the calcination process involved a high sintering temperature of 420 °C for 1 h, which is a drawback for this study. The electrochemical method has various advantages, including low temperature, precise control over composition, formation of uniform films, cost-effectiveness, direct deposition on substrate, in situ oxidation and selective deposition. Orimolade et al. [56] proposed the electrodeposition method followed by a calcination process for the preparation of Bi_2_WO_6_. Therefore, future research may focus on the fabrication of Bi_2_WO_6_ films on conductive substrates through the electrochemical method by avoiding the calcination process.

### 2.4. Sol–Gel Method

Previously, Zhang et al. [57] adopted the sol–gel method for the synthesis of Bi_2_WO_6_. In brief, bismuth nitrate and citric acid were dissolved in distilled water, stirring the reaction solution at 80 °C, and ethylene diaminetetraacetic acid (EDTA) ammonia solution was added, followed by the addition of ammonium tungstate. The obtained colorless transparent aqueous solution was evaporated at 80 °C and formed dark-colored polymeric precursors/citric gels. The calcination at 350 °C for 4 h yielded a Bi_2_WO_6_ powder sample. A further study indicated that the sol–gel method can be used for the preparation of Bi_2_WO_6_/graphene thin films, which exhibited excellent catalytic properties for nitric oxide oxidation [58]. Mesoporous Bi_2_WO_6_ sheets were also prepared by Han et al. [59] by adopting the sol–gel method. The synthesized material showed a high surface area of 36.7 m^2^ g^−1^, with a pore volume of 0.07 cm^3^ g^−1^. Therefore, Bi_2_WO_6_ displayed higher catalytic activity for photocatalytic applications. It is also understood that the sol–gel method also has numerous advantages, including homogeneous mixing, controlled composition, large-scale production, scalability, cost-effectiveness and coating capability.

### 2.5. Microwave Method

In 2023, the microwave method was adopted for the preparation of Bi_2_WO_6_ [60]. The microwave method involves the use of microwave irradiation to heat the precursor solution. A flower-like Bi_2_WO_6_ microstructure was also prepared using the rapid microwave-assisted method [61]. The authors also prepared a Bi_2_O_3_/Bi_2_WO_6_ composite using the microwave method. A Bi_2_WO_6_/Bi_2_S_3_ heterojunction was also developed by Liu et al. [62] for the reduction of chromium ions using the photocatalytic method. They suggested that the microwave method can also be used for the preparation of hybrid composite materials. It is also worth mentioning that the microwave method has several advantages, such as a shorter reaction time, fast heating process, uniform heating, and reproducibility. However, the large-scale production of Bi_2_WO_6_ via the microwave method is limited.

## 3. Progress in Electrochemical Sensors

**Figure 1:**
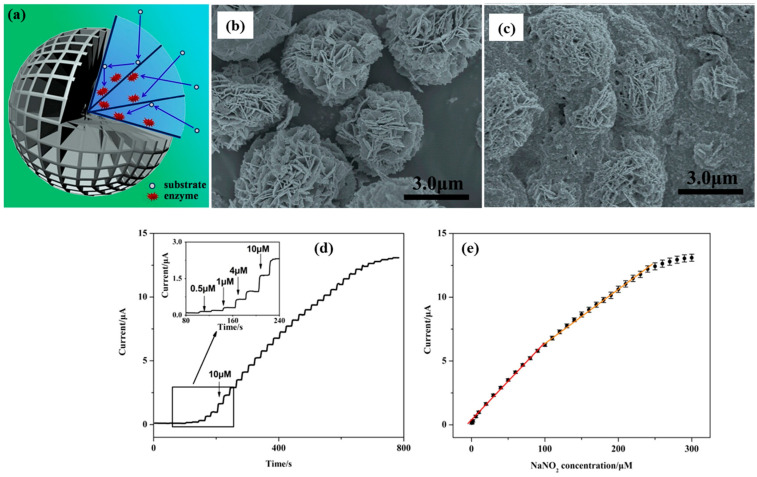
(**a**) Schematic description for 3D-Bi_2_WO_6_ MSs immobilizing HRP. SEM image of (**b**) 3D-Bi_2_WO_6_ MSs and (**c**) Nafion/HRP/3D-Bi_2_WO_6_ MSs. AMP response of the modified electrode for H_2_O_2_ sensing (**d**) and corresponding linear calibration plot (applied potential = −0.35 V) (**e**). Reproduced with permission [64].

Electrochemical sensors are the most promising next-generation devices, offering several advantages towards the monitoring of environmental pollutants and biomolecules. Electrochemical sensors can be utilized for environmental monitoring and clinical/healthcare systems. The selection of a suitable electrode modifier is of great significance for the development of highly sensitive and selective electrochemical sensors. In this context, a cost-effective and promising electrode material (Bi_2_WO_6_) was explored for electrochemical sensing applications. Peng et al. [63] explored the potential application of hydrothermally grown Bi_2_WO_6_ nanoplates (NPts) for the simultaneous detection of diethylstilbestrol (DES) and bisphenol A (BPA). The formation of the Bi_2_WO_6_ phase was authenticated using the X-ray diffraction (XRD) method, whereas its morphological features were evaluated via scanning electron microscopy (SEM). It is well understood that DES and BPA are environmental pollutants; therefore, the detection of DES and BPA is necessary. Electrochemical impedance spectroscopy (EIS)-based investigations revealed that a Bi_2_WO_6_ NPts-modified carbon paste electrode (CPE) exhibits a low charge transfer resistance (R_ct_) value compared to the bare CPE. It suggests that Bi_2_WO_6_ NPts-modified CPE possesses higher electrical conductivity and electrocatalytic properties, which may be ascribed to the presence of Bi_2_WO_6_ NPts on the CPE surface. The cyclic voltammetry (CV) results shows that diffusion plays a crucial role for the electrochemical determination of DES and BPA. Differential pulse voltametric (DPV) analysis further suggested that redox peak current responses for DES and BPA linearly increase with increasing concentration. Thus, these authors were able to obtain limits of detection (L_OD_) of 15 nM and 20 nM for the sensing of DES and BPA, respectively. The spike approach-based studies indicate that DES and BPA can be recovered from milk samples. Liu et al. [64] adopted the hydrothermal synthetic technique for the fabrication of three-dimensional (3D) Bi_2_WO_6_. The SEM results shows that the prepared Bi_2_WO_6_ is composed of flower-like microspheres (MSs), which were further used to immobilize horseradish peroxidase (HRP) for the construction of a hydrogen peroxide (H_2_O_2_) sensor. H_2_O_2_ is widely used in various industries, but its toxic effects on human health motivated the researcher to develop electrochemical sensors. The immobilization process is described in Figure 1a. The flower-like MSs structure of the prepared Bi_2_WO_6_ was confirmed through SEM analysis, as shown in Figure 1b. The immobilization of HRP with Bi_2_WO_6_ MSs was also confirmed, as seen in the SEM image (Figure 1c). The nafion/HRP/3D-Bi_2_WO_6_ was coated on a glassy carbon electrode (GCE) to determine the H_2_O_2_ using the amperometry (AMP) technique. The AMP response of the nafion/HRP/3D-Bi_2_WO_6_/GCE with the spike of different H_2_O_2_ concentrations is presented in Figure 1d. It was observed that the current response linearly increased with a spike in H_2_O_2_ (Figure 1e). The stability for 30 days was also checked for long-term applications, and further studies revealed good reproducibility for the detection of H_2_O_2_. The H_2_O_2_ recovery in real samples was also satisfactory, revealing the potential of the proposed nafion/HRP/3D-Bi_2_WO_6_/GCE for practical applications.

**Figure 2:**
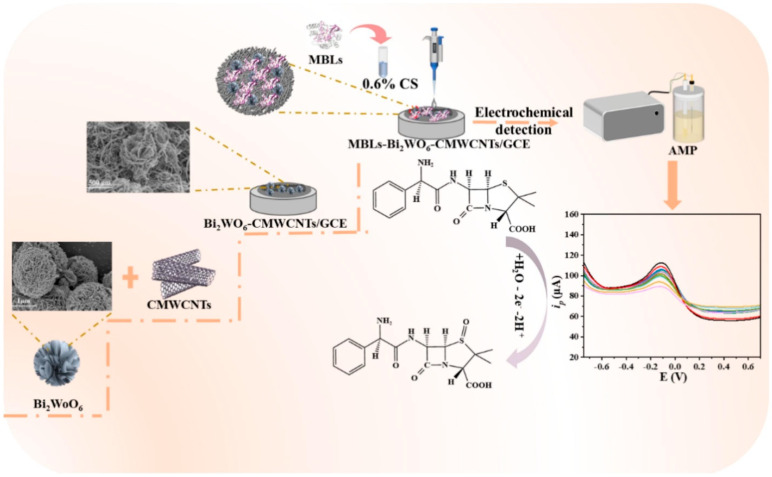
Schematic graph shows the fabrication of MBLs/CS/Bi_2_WO_6_/MWCNTs-COOH/GCE towards the determination of AMP. Reproduced with permission [70].

In other previous work [65], the voltametric determination of Sudan I was also reported using Bi_2_WO_6_ nanosheet (NS)-modified GCE. The formation of Bi_2_WO_6_ NS was checked using Raman spectroscopy, SEM, XRD, energy-dispersive X-ray spectroscopy (EDX) and Fourier transform infrared (FTIR) spectroscopy. The electrochemical oxidation of Sudan I involves a two-proton/two-electron transfer mechanism and diffusion-controlled process. The L_OD_ of 2 nM, sensitivity of 3.0563 µA µM^−1^ and linear range (L_R_) of 0.02 µM to 114.6 µM were achieved for Sudan I sensing. The authors also recovered Sudan I from food samples, including chili powder and apple juice. The dual-mode electrochemical/photoelectrochemical detection platform for hydrogen sulfide (H_2_S) was explored by Xu et al. [66]. In this context, a titanium dioxide (TiO_2_)-modified Bi_2_WO_6_/silver (Ag) heterojunction was developed for the reduction of H_2_O_2_. The proposed electrode exhibited an L_OD_ of 60 nM and L_R_ of 0.5 µM to 300 µM, with reasonably good stability and reproducibility. This electrode was also efficient and selective for the determination of H_2_O_2_ in the presence of many interfering substances, including selectivity, Na_2_SO_4_, Na_2_S_2_O_3_, NaHSO_4_, Na_2_SO_3_, ascorbic acid (AA), l-Cys, dopamine (DA), and 30 μM H_2_S. The presence of synergistic interactions may be responsible for the improved selectivity and electrochemical performance of the proposed H_2_O_2_ sensor. Hu et al. [67] developed a sulfamethoxazole (SMX) electrochemical sensor by exploring Br-terminated 2D Bi_2_WO_6_ as a sensing layer. The authors proposed that the prepared Br-terminated 2D Bi_2_WO_6_ can be explored in electrochemical sensing and photochemical water treatments. Vitamins are organic compounds and are essential for the operation of the human body. Vitamin deficiency may cause diseases in the human body. Riboflavin (RF), which is also called vitamin B2 (RF-7, 8-dimethyl 10 ribityl-isoalloxazine), plays a vital role in the development of flavin mononucleotide (FMN) and flavin coenzyme, comprising flavin adenine dinucleotides (FADs), which are important for tissue aeration. It is well known that RF can facilitate the transformation of fats, proteins, and carbohydrates to ATP. RF deficiency may cause biological problems such as burning and scorching of the eyes. Therefore, monitoring the level of RF is important. In this regard, Bi_2_WO_6_ was prepared, and its morphological characteristics were optimized by using sodium hydroxide (NaOH), polyvinylpyrrolidone (PVP) and a mixture of ternary solvents. The hollow spheres of Bi_2_WO_6_ were obtained by employing PVP + NaOH as a solvent [68]. The Bi_2_WO_6_ hollow sphere-modified GCE exhibited improved electro-oxidation of RF using the DPV technique. The fabricated RF sensor displayed a reasonable L_OD_ of 3.65 nM, sensitivity of 1.14 µA µM^−1^ cm^−2^ and L_R_ of 0.03 µM to 457 µM for the reduction of RF, whereas the oxidation process showed an L_OD_ of 8.9 nM with a sensitivity of 0.95 µA µM^−1^ cm^−2^. The presence of hollow spheres acted as a tunnel for the travel of electrons and ions, which enhanced the electrochemical sensing of RF. In another report [69], Bi_2_WO_6_ was decorated on a paraffin-deposited graphite electrode (PGE) for the electrochemical determination of AA. AA is an efficient antioxidant and plays a vital role in the human body. Accurately monitoring the level of AA is of great significance. Bi_2_WO_6_/PGE shows acceptable electrochemical performance for the detection of AA using CV and DPV techniques, and observations revealed that the fabricated electrode has higher electrochemical activity for AA detection at a pH of 7.0. The current response was also linearly increased, and an L_OD_ of 77.85 mM was obtained, with a sensitivity of 0.26 mM/mA. The L_OD_ of the Bi_2_WO_6_/PGE-based AA sensor needs to be further improved by introducing novel strategies or combining Bi_2_WO_6_ with other conductive and high-surface-area materials. The misuse of antibiotics such as ampicillin (AMP) is dangerous for humans as their residues can enter the human body through the ecological and food chains and can exert negative effects on human health. Tang et al. [70] reported the fabrication of a novel AMP sensor by exploring the electrochemical properties of a Bi_2_WO_6_-based composite. The hydrothermally prepared Bi_2_WO_6_ was combined with carboxylated multi-walled carbon nanotubes (MWCNTs-COOH) by employing the ultrasonication method. Other steps involved the immobilization of metal β-lactamases (MBLs) + chitosan (CS) to develop the MBLs/CS/Bi_2_WO_6_/MWCNTs-COOH/GCE for the determination of AMP (Figure 2). The constructed MBLs/CS/Bi_2_WO_6_/MWCNTs-COOH/GCE showed improved stability, reproducibility, repeatability, selectivity and acceptable recovery of AMP in real samples. The L_OD_ of 0.17 nM and two L_R_ of 0.0007 to 0.05 µM and 0.1 to 10 µM were observed for AMP detection. The sensing mechanism for the electrochemical detection of AMP is explained in Figure 2.

**Figure 3:**
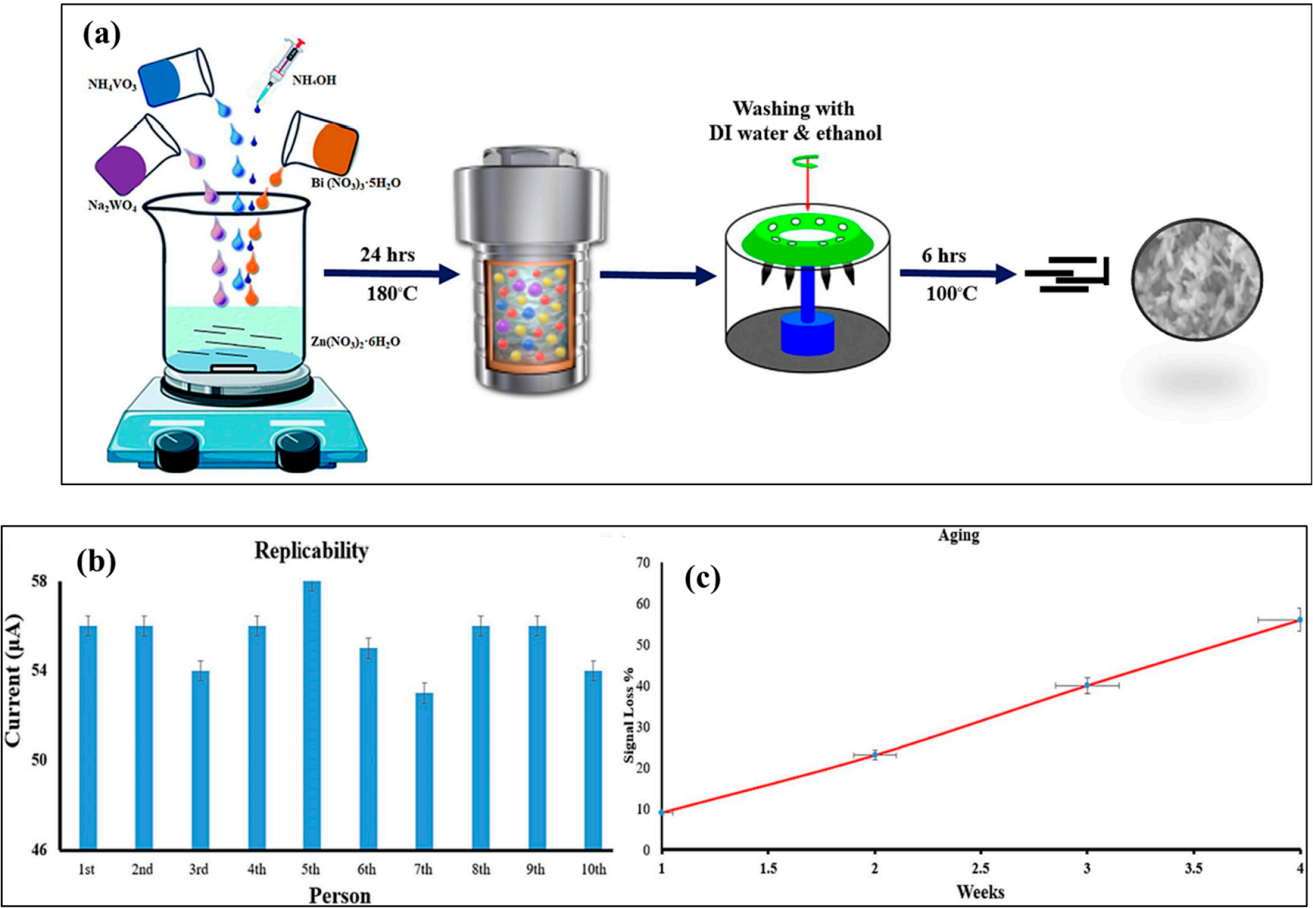
(**a**) Schematic graph for the preparation of Zn_3_(VO_4_)_2_/Bi_2_WO_6_ composite. (**b**) Replicability and (**c**) stability of Zn_3_(VO_4_)_2_/Bi_2_WO_6_-based cotinine sensor. Reproduced with permission [72].

Another study reported the benign wet-chemical co-precipitation method for the preparation of Bi_2_WO_6_ [71]. The prepared Bi_2_WO_6_ was characterized by XRD, which confirmed the formation of the desired product and matched with JCPDS number 73-1126. The authors observed that XRD analysis revealed the formation of orthorhombic Bi_2_WO_6_. The transmission electron microscopy (TEM) analysis indicated the presence of the polycrystalline nature of the prepared orthorhombic Bi_2_WO_6_. In further studies, it was observed that the Bi_2_WO_6_-modified electrode has relatively higher electrical conductivity compared to the bare electrode. The authors explored the potential of the Bi_2_WO_6_-modified electrode for the simultaneous determination of hydroquinone and resorcinol (HQ and RS) using the CV and DPV methods. The pH of the analyte solution was also optimized, and it was observed that the fabricated electrode is highly active at a pH of 7.0. In high basic or high acidic pH conditions, no significant peak was observed for the detection of HQ and RS. This sensor was also found to be highly stable, selective, and reproducible for the determination of HQ and RS. The real sample studies in tap water and ointment suggested its significance for the real-time monitoring of HQ and RS. Previously, a zinc vanadate-modified Bi_2_WO_6_ (Zn_3_(VO_4_)_2_/Bi_2_WO_6_) composite was fabricated using the hydrothermal method, as shown in Figure 3a [72]. The prepared Zn_3_(VO_4_)_2_/Bi_2_WO_6_ shows decent crystalline nature and was found to be in agreement with JCPDS number 24-1482 and 39-0256 for Zn_3_(VO_4_)_2_ and Bi_2_WO_6_, respectively. It was also observed from XRD analysis that Zn_3_(VO_4_)_2_ has a monoclinic phase, whereas Bi_2_WO_6_ possesses an orthorhombic crystal structure. The Zn_3_(VO_4_)_2_/Bi_2_WO_6-_modified electrode demonstrated an L_OD_ of 1 pM and L_R_ of 1 nM to 5 µM for the determination of cotinine. The proposed cotinine sensor also showed good replicability and stability, as shown in Figure 3b and Figure 3c, respectively.

**Figure 4:**
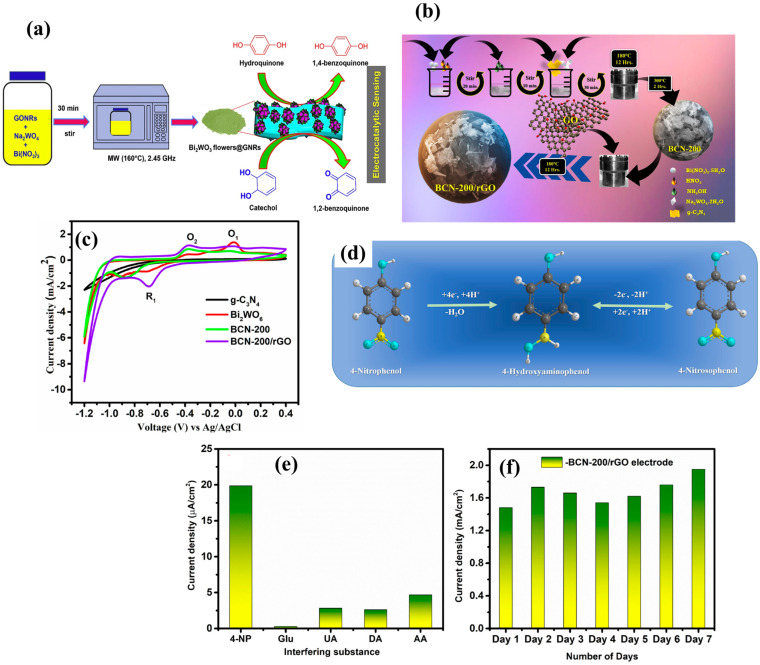
(**a**) Schematic representation of the fabrication of Bi_2_WO_6_@GNRs for HQ and CC sensing. (**b**) Schematic representation of the fabrication of BCN-200/rGO. (**c**) CVs of g-CN, Bi_2_WO_6_, BCN-200 and BCN-200/rGO for 4-NP sensing. (**d**) Mechanism for 4-NP detection. (**e**) Selectivity and (**f**) stability test. Reproduced with permission [76,77].

A single-step hydrothermal method was explored for the fabrication of a morphologically engineered biomimetic 3D biconcave-shaped Bi_2_WO_6_ [73]. The authors used aluminum (Al) foil as a substrate to develop the Bi_2_WO_6_-based nicotine electrochemical sensor. The Bi_2_WO_6_-modified Al foil-based nicotine sensor exhibited excellent selectivity, L_OD_ of 0.9 nM, sensitivity of 83.23 μA/(μM cm^2^), and acceptable stability/reproducibility. Another study reported the fabrication of a photoelectrochemical DA sensor using the benign approach [74]. In this connection, a cobalt tetraaminophthalocyanine covalently linked graphene oxide (CoTAPc-GO) composite was fabricated using simple strategies. Furthermore, CoTAPc-GO and Cu-doped Bi_2_WO_6_ microflowers (MFs) were combined by employing the ultrasonication method. The obtained material (CoTAPc-GO/Cu-Bi_2_WO_6_) was coated on an indium tin oxide (ITO) surface, and its sensing activity for DA was evaluated. The authors obtained an L_OD_ of 7.2 nM, with two L_R_ of 0.05 µM to 5 µM and 5 µM to 250 µM for the detection of DA. Xu et al. [75] reported a photoelectrochemical immunosensor for the determination of amyloid beta (Aβ). The flower-like amorphous structure of Bi_2_WO_6_ was obtained, which was further combined with Mn^2+^-doped cadmium selenide (CdSe). The obtained results exhibited a reasonable L_OD_ of 0.068 pg/mL with L_R_ of 0.2 pg/mL to 50 ng/mL. The flower-like Bi_2_WO_6_ was decorated on graphene nanoribbons (GNRs) by employing the microwave synthesis method [76]. The fabrication procedure for Bi_2_WO_6_@GNRs is depicted in Figure 4a. The Raman and X-ray photoelectron spectroscopy (XPS) studies confirmed the presence of Bi_2_WO_6_ on the GNR surface. Further studies revealed that a Bi_2_WO_6_@GNR-modified screen-printed electrode (SPE) has a good selective nature for the determination of HQ and CC. The impedance studies indicate the presence of enhanced electrical conductivity of the constructed Bi_2_WO_6_@GNRs/SPE. Therefore, Bi_2_WO_6_@GNRs/SPE shows an L_OD_ of 5.31 nM and 7.24 nM for CC and HQ detection, respectively. The proposed sensor was also effective for the detection of CC and HQ in a water sample and face cream sample, respectively. In addition, the proposed sensor exhibited decent selectivity for the determination of CC and HQ in the presence of various interfering substances (Ni^+^, Cu^2+^, Cr^+^, Na^+^, Zn^2+^, Cl^−^, Hg+, Cd^+^, DA, hydrazine (Hz), nitrobenzene (NBz), 4-nitrophenol (4-NP), 4-aminophenol (4-AP), 2-aminophenol (2-AP), glucose (glu), nitrite, resorcinol (RS), epinephrine, AA, vitamin B_6_ and vitamin B_9_). The enhanced performance of the Bi_2_WO_6_@GNRs/SPE was ascribed to the presence of synergistic interactions and the high surface area of the electrode material. Verma et al. [77] prepared a plate-like Bi_2_WO_6_-modified mesh-like graphitic carbon nitride (g-CN) composite, and rGO was loaded into this prepared hybrid material using the hydrothermal-assisted synthesis method. The authors tuned the percentage of g-CN, and the optimized sample BCN-200/rGO (Bi_2_WO_6_/g-CN-200 mg/rGO) exhibited enhanced catalytic properties. The formation of the BCN-200/rGO is shown in Figure 4b. The obtained Bi_2_WO_6_/g-CN/rGO composite was explored for the fabrication of an electrochemical sensor towards the detection of 4-NP. The CVs of the g-CN, Bi_2_WO_6_, BCN-200 and BCN-200/rGO-modified GCE are shown in Figure 4c.

It can be seen that BCN-200/rGO/GCE has a higher redox peak current response for the detection of 4-NP. The mechanism involved in the sensing of 4-NP is shown in Figure 4d. The BCN-200/rGO-modified GCE also showed excellent selectivity and stability for the sensing of 4-NP, as shown in Figure 4e and Figure 4f, respectively. The presence of synergy in the Bi_2_WO_6_, g-CN, and rGO enhanced the catalytic behavior of the BCN-200/rGO-modified GCE. Additionally, the fabricated 4-NP sensor delivered an L_OD_ of 14 nM, L_R_ of 0.2 μM to 100 μM and sensitivity of 12.86 μA.μM^−1^.cm^−2^. It is well known that 4-Nitroquinoline N-oxide (4-NQO) is an important tumorigenic organic compound, which has adverse effects on the human body. Thus, in a previous report, a Bi_2_WO_6_ nanosphere-decorated rGO composite was prepared using the sonication method [78]. The Bi_2_WO_6_/rGO composite was further deposited on the GCE surface and its electrochemical activities for the detection of 4-NQO using the AMP method. The proposed electrode shows an L_OD_ of 6.11 nM and excellent detection of 4-NQO in urine and human blood samples. The plasmonic Ag-induced metallic Ag Bi_2_WO_6_/TiO_2_ composite was developed for oxygen/hydrogen evolution and DA electrochemical sensing applications [79]. The CV results show that the current response linearly increases with an increasing concentration of DA. Due to the multifunctional applications of the proposed material in a single report, the authors did not significantly focus on the sensing of DA. Thus, more in-depth studies are required to explore the potential of the above-proposed electrode material for DA sensing applications. In recent years, MXene-based materials have been widely used to enhance the physicochemical properties of seminconducting metal oxides and metal sulfides, etc. The incorporation of MXene materials enhanced the electrical conductivity of the resulting composite materials. In this context, niobium carbide (Nb_4_C_3_T_x_) MXene was incorporated with Bi_2_WO_6_ (as first layer), and salmon ds-DNA was engineered as a second layer/biological recognition element on a conductive substrate [80]. Thus, these authors obtained a decent L_OD_ of 2.8 nM, L_R_ of 0.01 µM to 100 µM, and acceptable recovery of 98.7% to 103.6% for the determination of pemetrexed using Bi_2_WO_6_/Nb_4_C_3_Tx/ds-DNA/SPE. Another study [81] reported a novel sandwich-type SARS-CoV-2 (COVID-19) nucleocapsid protein immunosensor by employing simple strategies. The Bi_2_WO_6_/bismuth sulfide (Bi_2_S_3_) composite was utilized as an electrode platform, whereas g-CN/Au NPs/tungsten trioxide (WO_3_) was adopted for signal amplification. The proposed sensor delivered an L_OD_ of fg/mL. Nitrite has a negative influence on human health and the environment due to its toxic properties. The determination of nitrite is necessary, and a previous report indicated that Co phthalocyanine (CoPc) NPs were covalently linked to Bi_2_WO_6_ and nickel tungstate (NiWO_4_), which can used as sensing layers for the determination of nitrite [82]. The authors observed that CoPc covalently linked to Bi_2_WO_6_-modified GCE shows better performance compared to the CoPc covalently linked to NiWO_4_-modified GCE. An interesting LOD of 0.063 µM to 1.7 µM was observed for the detection of nitrite. Kanamycin (KAN) is a well-known amino glycoside, which has serious negative impacts on human health. In this regard, it is useful to develop the KAN sensor by exploring Bi_2_WO_6_/Ti_3_C_2_ MXene QDs [83]. The electrostatically driven hydrothermal-assisted-formed Bi_2_WO_6_/Ti_3_C_2_ MXene QD composite was adopted as a sensing material. The proposed KAN sensor exhibited recoveries in the range of 94.2% to 101.3%, L_OD_ of 0.025 nM, and L_R_ of 0.01 nM to 50 nM. The dual 3D structural Bi_2_WO_6_/graphene hydrogel (GH)-modified molecularly imprinted polymer (MIP)-based electrode was developed for the detection of 4-NP in PM_2.5_ [84]. The fabricated MIPs/Bi_2_WO_6_@GH/ITO electrode had an L_OD_ of 5.78 × 10^−13^ M and L_R_ of 5.0 × 10^−12^ to 1.0 × 10^−7^ M for the determination of 4-NP. This enhanced electrochemical performance of the proposed electrode was attributed to the 3D flower-like morphology of Bi_2_WO_6_ and porous network of GH. It is proven that hexavalent chromium (Cr) has toxic effects, and exposure to Cr via ingestion or inhalation may cause allergies, affect internal organs, and contribute to cancer. Therefore, it is really of great significance to develop an electrochemical sensor for the monitoring of Cr. In this connection, the solvothermal method was adopted for the preparation of a β-Bi_2_O_3_/Bi_2_WO_6_/*meso*-tetraphenylporphyrin (H_2_TPP) composite on a fluorine-doped tin oxide (FTO) substrate [85]. It was observed that the presence of H_2_TPP enhances the active site density and provides an efficient surface area for effective adsorption by providing pyrrolic and pyridinic-N atoms to β-Bi_2_O_3_/Bi_2_WO_6_/H_2_TPP. The β-Bi_2_O_3_/Bi_2_WO_6_/5 wt % H_2_TPP exhibited excellent cyclic stability, sensitivity, reproducibility and an L_OD_ of 8 nM. The electrochemical sensing performances of the various reported electrochemical sensors are summarized in Table 1.

It can be summarized that the above-discussed electrochemical sensors demonstrated promising electrochemical performance for the determination of various environmental pollutants. It was observed that pristine Bi_2_WO_6_ exhibits moderate electrochemical performance in terms of sensitivity. In contrast, the electrochemical performance of pristine Bi_2_WO_6_ was significantly enhanced by combining it with conductive materials, such as rGO, MXenes (Nb_4_C_3_Tx, Ti_3_C_2_), MWCNTs, g-C_3_N_4_, NiO, or Ag NPs. It was observed that Bi_2_WO_6_/MWCNTs/MBLs/CS exhibits the lowest LOD of 0.17 nM for ampicillin detection due to the presence of synergistic interactions such as high surface area and more abundant active sites. On the other side, the Bi_2_WO_6_/g-C_3_N_4_/rGO composite-based electrode demonstrates enhanced sensitivity of 12.86 μA μM^−1^ cm^−2^ for 4-NP, which can be assigned to the high electron mobility and surface area of the electrode material. It can be understood that Bi_2_WO_6_-based hybrid composites with MXenes and carbonaceous materials (rGO or CNTs) are promising electrode materials compared to the other hybrid composites for electrochemical sensing applications.

## 4. Progress in SCs

The design and fabrication of novel electrode materials are of great importance for SCs. Bi_2_WO_6_-based composites were significantly explored for the construction of SCs. Herein, we compile the progress in the preparation of Bi_2_WO_6_-based materials for the development of next-generation SCs.

### 4.1. Bi_2_WO_6_-Based Materials for SCs

**Figure 5:**
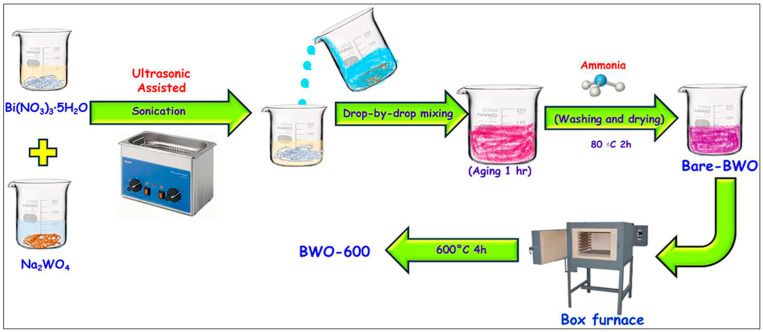
Schematic graph presenting synthetic procedure for BWO-600. Reproduced with permission under license CC-BY-NC-ND 4.0 [91].

In a previous report, Bi_2_WO_6_ NPs were synthesized via the sonochemical approach to explore an ecofriendly and cost-effective SC device [86]. The electrochemical performance of the Bi_2_WO_6_ NPs was evaluated in different aqueous electrolytes (1 M NaOH, 1 M sodium sulfate (Na_2_SO_4_), and 1 M lithium hydroxide (LiOH)). It was revealed that the KOH electrolyte was effective compared to the other electrolytes. Thus, the authors also used 1 M and 6 M KOH solutions for further electrochemical studies with higher ionic mobility, smaller hydration sphere radius and lower equivalent series resistance. The galvanostatic charge–discharge (GCD) studies show a specific capacitance of 608 F/g, which can be obtained for Bi_2_WO_6_ in 1 M KOH at a current density of 0.5 mA/cm^2^. The specific capacitance of 304 F/g at 3 mA/cm^2^ was also observed in a 1 M KOH electrolyte system. This study suggested that Bi_2_WO_6_ NPs can be explored as negative electrode modifiers for SCs in a 1 M KOH electrolyte. In another study [87], Bi_2_WO_6_ was also synthesized using the hydrothermal method. The authors used different times (3, 6, 12 and 24 h) for the preparation of Bi_2_WO_6_. The synthesized Bi_2_WO_6_ samples at 3, 6, 12 and 24 h were named BWO1, BWO2, BWO3 and BWO4, respectively. The optimized studies show that Bi_2_WO_6_-based SCs exhibit a specific capacitance of 363 F/g in neutral electrolytes and 5000 cyclic stabilities. The 3D Bi_2_WO_6_ globules were vertically grown in nickel foam (NF) electrodes using benign conditions [88]. The 3D Bi_2_WO_6_-modified NF electrode was utilized as a negative electrode, and areal capacitance of 1.120 F/cm^3^ was observed at a current density of 1.5 mA/cm^2^ with excellent cyclic stability of 1000. Shembade et al. [89] reported the hydrothermal preparation of mesoporous spherical nanoflower-like Bi_2_WO_6_. The Bi_2_WO_6_ was deposited on NF, and its electrochemical performance for SCs was examined using CV and GCD. It was found that Bi_2_WO_6_ NFs exhibit the presence of an orthorhombic phase in which atoms are present in a regular arrangement. The optimized electrode (Bi_2_WO_6_/NF-2 = BWO-2) showed a specific capacitance of 647 F/g in a 1 M KOH electrolyte, including stability of 5000 cycles. In another study, various nanostructures of Bi_2_WO_6_ were obtained by exploring hydrothermal treatment under various pH conditions [90]. pH 1-, 7- and 11-based synthesis exhibits the formation of Bi_2_WO_6_ with flower-like hierarchical structures (average diameter = 7 µm), irregular flake-like structures (average thickness = 90 nm) and uniform spherical structures (average size = 85 nm), respectively. The flower-like hierarchical, flake-like and spherical-shaped Bi_2_WO_6_ demonstrated specific capacitance of 255 F/g, 214 F/g, and 412 F/g, respectively. It can be noted that Bi_2_WO_6_ spheres exhibited the highest electrochemical performance for SCs. Karnan et al. [91] pioneered one-pot preparation of Bi_2_WO_6_ nanostructures for SCs. A benign ultrasonic-assisted synthesis method was adopted to fabricate bare Bi_2_WO_6_ (BWO-bare). The authors also heated Bi_2_WO_6_ at 600 °C, which can be labeled as BWO-600. Figure 5 illustrates the formation of BWO-600, and the obtained material was characterized by XRD and SEM to investigate its phase purity and morphological features. The SEM analysis revealed that BWO-600 consists of a nanostructure with a diameter of 50 to 310 nm. In contrast, BWO-bare does not show evident clusters. Under a 6 M KOH electrolyte system, the BWO-600-based electrode shows specific capacitance of 614 C/g in a three-electrode system, whereas the specific capacity of 376 C/g was observed in asymmetric SCs.

Other work adopted the solution combustion method for the preparation of Bi_2_WO_6_ NPs [92]. The authors used jackfruit extract as fuel in the synthesis of Bi_2_WO_6_ NPs. Nithya et al. [93] proposed the fabrication of the polyvinylpyrrolidone (PVP)-assisted synthesis of Bi_2_WO_6_ NPs through the sonochemical method. The concentration of the PVP was tuned in the range of 0.05 to 0.2 g. The optimized results for PVP-assisted Bi_2_WO_6_ demonstrated an acceptable specific capacitance value of 462 F/g, which is higher than pure Bi_2_WO_6_ (304 F/g) at a current density of 3 mA/cm^2^. In another study, Nithya et al. [94] also proposed the preparation of the surfactant-assisted sonochemical synthesis of Bi_2_WO_6_ NPs, which exhibited specific capacitance of 708 F/g under the optimized conditions.

### 4.2. Bi_2_WO_6_-Based Compoiste Materials for SCs

**Figure 6:**
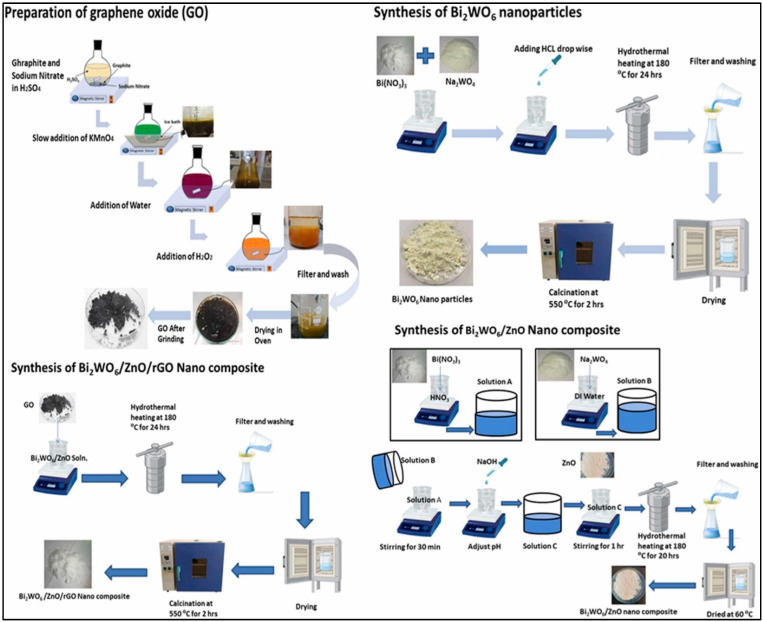
Schematic representation of the formation of the proposed Bi_2_WO_6_/ZnO/rGO composite. Reproduced with permission [97].

The supercritical water method was also adopted for the fabrication of a Bi_2_WO_6_/rGO hierarchical composite [95]. The supercritical water approach requires much less time to complete the reaction for the preparation of the Bi_2_WO_6_/rGO composite. The proposed electrode material exhibited specific capacity of 1158 C/g at 3 A/g in a 6 M KOH electrolyte. A novel electrode material, i.e., a bismuth vanadate (BiVO_4_)/Bi_2_WO_6_ microsphere composite, was also prepared using the microwave-assisted hydrothermal method [96]. It was stated that decoration of the BiVO_4_ NPs onto the Bi_2_WO_6_ NSs may prevent the restacking of B_i2_WO_6_ NSs in a disorganized manner and facilitate the formation of a mesoporous BiVO_4_/Bi_2_WO_6_ hierarchical microsphere composite. It was also found that the prepared BiVO_4_/Bi_2_WO_6_ hierarchical microsphere composite has higher structural stability, larger surface area and enhanced electron/ion migration. Thus, due to the above promising properties, an interesting specific capacitance of 616.8 F/g was obtained at 1 A/g and a stability of 4000 cycles with 87% retention using BiVO_4_/Bi_2_WO_6_ hierarchical microspheres as an electrode material. The synthesis of the Bi_2_WO_6_/ZnO/rGO composite is described in Figure 6 [97]. The Bi_2_WO_6_/ZnO/rGO composite was prepared via multiple steps. It was believed that the proposed ternary material may be a promising electrode modifier for SC applications. The ratio of the rGO was optimized to enhance the electrochemical activity of the Bi_2_WO_6_/ZnO/rGO composite for SC applications. The authors achieved a specific capacitance of 254.34 F/g at 2 A/g under optimized conditions. The presence of decent electrical conductivity, improved electrochemical activity and synergistic interactions enhanced the performance of the Bi_2_WO_6_/ZnO/rGO composite-based SCs.

**Figure 7:**
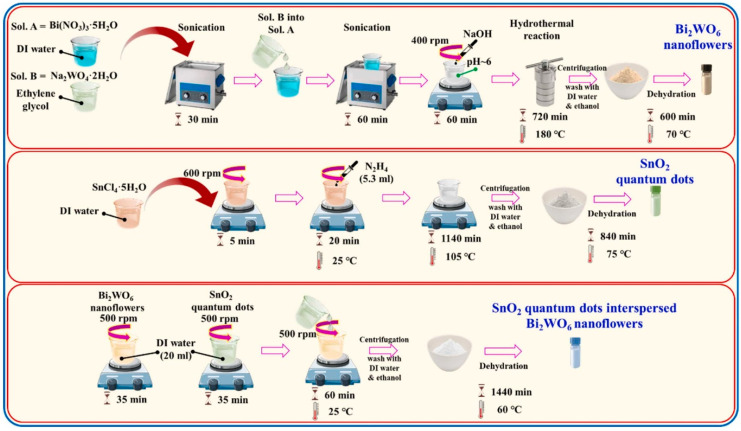
Schematic graph shows the fabrication of SnO_2_-Bi_2_WO_6_ NFs. Reproduced with permission [99].

Qadeer et al. [98] proposed that the electrochemical properties of the pristine Bi_2_WO_6_ can be further improved by combining it with titanium disulfide (TiS_2_). Therefore, the Bi_2_WO_6_/TiS_2_ composite was obtained via the hydrothermal method. The morphological characteristic properties of the Bi_2_WO_6_/TiS_2_ composite were checked using SEM technique, which revealed that TiS_2_ nanoplates are uniformly dispersed across the hierarchically opened surface of Bi_2_WO_6_. Thus, it was observed that the resulting Bi_2_WO_6_/TiS_2_ composite exhibited improved porosity and specific surface area, which may facilitate the electron and ion transport. The GCD studies displayed specific capacitance of 453.3 F/g, 523.7 F/g and 1153.3 F/g for TiS_2_, Bi_2_WO_6_, and Bi_2_WO_6_/TiS_2_ composites, respectively. Another previous study proposed the incorporation of tin oxide (SnO_2_) to the Bi_2_WO_6_ nanoflowers (NFs) using simple strategies, as shown in Figure 7 [99].

**Figure 8:**
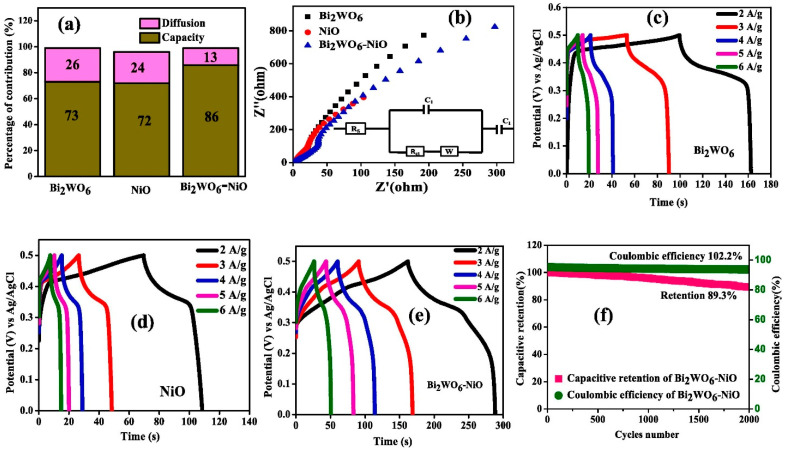
(**a**) Capacitive diffusion and (**b**) Nyquist plots. GCD curves of (**c**) Bi_2_WO_6_, NiO (**d**) and (**e**) Bi_2_WO_6_/NiO composite. (**f**) Capacitive retention/coulombic efficiency of Bi_2_WO_6_/NiO composite. Reproduced with permission [100].

The SnO_2_ QD-interspersed Bi_2_WO_6_ NFs were utilized as an electrode material, with a specific capacitance of 812 and 741 F/g at 7 A/g for Bi_2_WO_6_/SnO_2_ QDs and Bi_2_WO_6_-based electrodes, respectively. The improved specific capacitance for the Bi_2_WO_6_/SnO_2_ QD-based electrode may be ascribed to the quantum dot size of SnO_2_ and the presence of synergistic interactions. This material offers great potential for SC applications. Nickel oxide (NiO) is a promising semiconducting metal oxide, with reasonably good electrochemical properties. The Bi_2_WO_6_/NiO composite was adopted as an electrode modifier for SCs [100]. The capacitive diffusion and Nyquist plots of Bi_2_WO_6_, NiO and Bi_2_WO_6_/NiO composite-based electrodes are shown in Figure 8a and Figure 8b, respectively. It can be seen that the Bi_2_WO_6_/NiO composite has decent electrical conductivity. The GCD curves of the Bi_2_WO_6_, NiO and Bi_2_WO_6_/NiO composite at different current densities are depicted in Figure 8c, Figure 8d and Figure 8e, respectively. It can be observed that the prepared materials have higher specific capacity at 2 A/g. The highest specific capacitance for the Bi_2_WO_6_/NiO composite-based electrode was attributed to the presence of synergism in the hybrid composite material. The capacitive retention/coulombic efficiency of the Bi_2_WO_6_/NiO composite is shown in Figure 8f. Thus, it is also suggested that the Bi_2_WO_6_/NiO composite has decent cyclic stability of 2000 for SCs.

**Figure 9:**
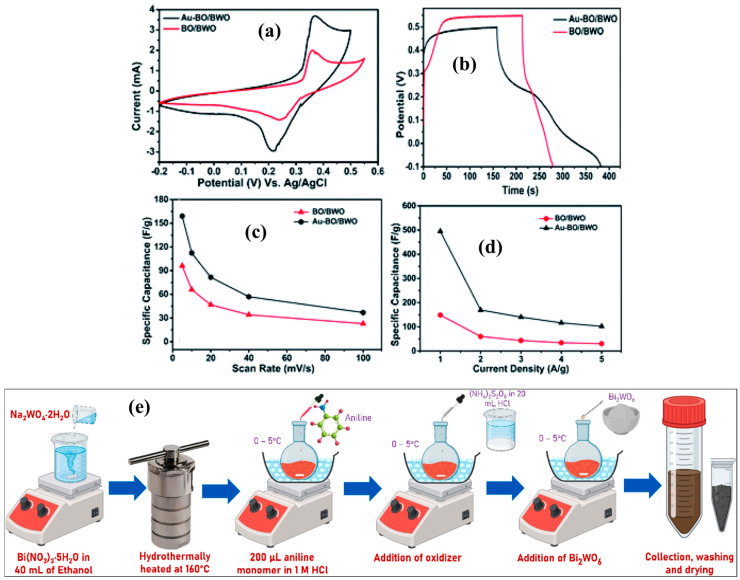
(**a**) CV curves of BO/BWO and Au-BO/BWO at scan rate of 10 mV/s. (**b**) GCE curves of BO/BWO and Au-BO/BWO at 1 A/g. (**c**) Specific capacitance versus scan rate and (**d**) specific capacitance versus current density graphs. Reproduced with permission [109]. (**e**) Schematic picture shows the preparation of BW-BIC-PANI composite. Reproduced with permission [110].

Zheng et al. [101] proposed a benign one-pot hydrothermal method for the fabrication of a 3D porous Bi_2_WO_6_/rGO hydrogel composite. The electrochemical studies revealed that the 3D porous Bi_2_WO_6_/rGO hydrogel composite has higher capacitive properties and demonstrates specific capacitance of 268.7 F/g at an applied current density of 0.75 A/g. This material also displayed robust stability of 1000 cycles at 3 A/g, with capacitance retention of 81%. This improved performance may be due to the synergistic interactions, unique 3D porous rGO hydrogel-supported Bi_2_WO_6_ architecture, which provided good electrical conductivity and more channels for ion diffusion. Zhang et al. [102] also adopted a nano Bi_2_WO_6_-decorated rGO composite via the hydrothermal method. The proposed Bi_2_WO_6_/rGO composite shows improved electrical conductivity and increased charge transfer channels. Thus, it is expected that the presence of these properties may facilitate the electron transfer, and Bi (III) may oxidize to Bi (IV), which provide higher specific capacitance and stability. Therefore, the authors achieved a specific capacitance of 922 F/g at 3 A/g, which is higher than pristine Bi_2_WO_6_ (574 F/g at 2 A/g). The Al-based MOF (Materiaux De Lavoisier = MIL)-53 was prepared via the solvothermal method [103]. It is observed from these studies that MIL-53 (Al) has low conductivity, which restricts its potential applications in energy storage systems. Therefore, MIL-53 (Al) was incorporated with Bi_2_WO_6,_ and its properties were evaluated via the CV, EIS and GCD methods. The MIL-53 (Al)/Bi_2_WO_6_-based electrode displayed a larger surface area and improved conductive nature. Therefore, an enhanced specific capacitance value of 220 F/g was observed at 0.5 A/g in the 3 M KOH electrolyte with excellent cyclic stability (5000 cycles; retention capacitance = 78%). Sheoran et al. [104] reported a novel electrode modifier comprising a carbon black (CB)-anchored bismuth tungstate-aniline complex composite ((Bi_2_(WO_4_)_3_/Aniline/CB) (BTACB)). The authors reported a specific capacitance of 306 F/g at 1 A/g, which can be attributed to the synergism between aniline complex, CB and bismuth tungstate, which provide larger surface area and acceptable conductivity. A sandwich-like heterostructure of the Bi_2_WO_6_/g-CN composite was developed using benign approaches [105]. This fabricated Bi_2_WO_6_/g-CN composite was adopted as an electrode modifier for energy storage applications, and the obtained results showed specific capacitance of 158 mF/g at 1 mA/cm^2^. It is well understood that 3D porous structures may promote the electron transport process and improve the electrochemical capacitive properties of the SCs. In this regard, the 3D aerogel/poriferous structure of Bi_2_WO_6_ sheets was combined with graphene NSs via benign hydrothermal treatment [106]. It was stated that the 3D multi-hole structure of the prepared hybrid aerogel may provide high surface area and promote electron transport and ion transmission. This may decrease the internal electrical resistance and enhance the capacitive properties of the fabricated electrode. The Bi_2_WO_6_/graphene aerogel composite demonstrated specific capacitance of 714 F/g at 4 A/g. In another previous report [107], MIL-53 (Al) and Bi_2_WO_6_ were obtained via the hydrothermal route, whereas the MIL-53 (Al)/Bi_2_WO_6_ composite was fabricated using the impregnation method. Due to the lower equivalent series resistance and synergistic interactions, the MIL-53 (Al)/Bi_2_WO_6_ composite demonstrated specific capacitance of 554 F/g at 0.5 A/g. Due to the high surface area and high porosity, copper MOF (Cu-MOF) has been utilized in the fabrication of hybrid materials for SCs. In this connection, a Cu-MOF/Bi_2_WO_6_ binary hybrid composite was developed through the solvothermal method [108]. The weight percentage was varied to optimize the electrochemical performance of the Cu-MOF/Bi_2_WO_6_ binary hybrid composite for SCs. The samples with different weight percentages were labeled as CuBW20, CuBW50 and CuBW80. 3 M KOH was explored as a suitable electrolyte, and CuBW80 demonstrated excellent specific capacitance of 1137 F/g, specific power of 4000 W/kg, specific energy of 11 Wh/kg at a current density of 0.5 A/g. This optimized electrode material was also suitable for stability of 1000 cycles. In another study [109], Bi_2_O_3_/Bi_2_WO_6_ (BO/BWO) and Au-decorated Bi_2_O_3_/Bi_2_WO_6_ (Au-BO/BWO) composites were developed through the hydrothermal method. The electrochemical performance of BO/BWO and Au-BO/BWO was evaluated via CV analysis, as shown in Figure 9a. It can be seen that A-BO/BWO displayed higher electrochemical activity compared to BO/BWO. Similarly, A-BO/BWO showed improved capacitance and suggested its potential for SCs (Figure 9b). The specific capacitance versus scan rate graph is shown in Figure 9c, whereas the specific capacitance versus current density graph is depicted in Figure 9d. The BO/BWO-based electrode demonstrated specific capacitance of 148.81 F/g, whereas the highest specific capacity of 495.05 F/g for the A-BO/BWO-based electrode was obtained at 1 A/g in a 1 M KOH electrolyte system. The improved specific capacity of the A-BO/BWO-based electrode may be attributed to the presence of Au NPs, which improved electrical conductivity and decent electrical contacts between the electrolyte and electrode, which may have improved the effective surface area. The multi-functional polyaniline (PANI)-wrapped Bi_2_WO_6_ 2D nanoplate/BiOCl (BW-BIC-PANI) composite was also prepared by Kavinkumar et al. [110].

The fabrication of the BW-BIC-PANI composite via the hydrothermal method followed by the chemical oxidative polymerization method is shown in Figure 9e. The authors proposed that a specific capacitance of 156 F/g can be obtained at 1 A/g by employing a BW-BIC-PANI_200_-based electrode. The presence of synergistic interactions enhances the electrochemical capacitive properties of the BW-BIC-PANI_200_ for SCs. The hydrothermal method was also utilized for the preparation of Ag-doped Bi_2_WO_6_ (ABW) [111]. Furthermore, ABW was incorporated with MXene to fabricate the ABW@MXene composite via the sonication method. The specific capacitances of 870 F/g and 1420 F/g for the ABW@MXene-based electrode were observed using CV and GCD, respectively. In contrast, MXene exhibited a specific capacitance value of 200 F/g and 413 F/g using CV and GCD, respectively. The improved performance for ABW@MXene was attributed to the presence of synergistic interactions between ABW and MXene. Liu et al. [112] prepared Bi_2_S_3_ NFs, which comprised 1D NRs. The Bi_2_S_3_ NFs were obtained through a topotactic transformation approach. The Bi_2_WO_6_ NFs were used as a precursor template for the preparation of Bi_2_S_3_ NFs. The Bi_2_WO_6-_derived Bi_2_S_3_ NFs exhibited decent specific capacitance and electrochemical stability for SCs. The electrochemical capacitive performance of the Bi_2_WO_6-based mat_erials is summarized in Table 2.

## 5. Conclusions, Limitations and Perspectives

It is concluded that Bi_2_WO_6_ has been widely used for the fabrication of working electrodes for electrochemical sensing and SC applications. Bi_2_WO_6_ has low conductive properties, which can be enhanced by preparing Bi_2_WO_6_-based hybrid composites with conductive materials, such as carbon-based materials or MXenes. The Bi_2_WO_6_-based composites exhibited excellent sensing performance for the detection of various analytes, including DES, H_2_O_2_, H_2_S, Sudan I, ampicillin, 4-NP, pemetrexed, etc. It was suggested in previous reports that DPV is the most widely used sensing technique for the determination of environmental pollutants. Bi_2_WO_6_ may exhibit a smaller hydration sphere radius, higher ionic mobility, and lower equivalent series resistance, which benefit researchers by producing a higher specific capacity for SCs. The presence of Bi^3+^ with 6s^2^ lone pair electrons may improve the abundant active sites and electronic conductivity. Thus, Bi_2_WO_6_-based composites also exhibited excellent specific capacitance for SC applications. Despite several advantages, some challenges exist, which are mentioned below.

- Pristine Bi_2_WO_6_ suffers from low conductivity, which needs to be addressed.
- Although several efforts were made to enhance the conductivity of Bi_2_WO_6_ by combining it with conductive supports such as MXene or rGO, synthetic procedures are not cost-effective for practical applications.
- DPV is a more sensitive technique, but, in some cases, the presence of similar interfering substances affects the selectivity of electrochemical sensors.
- Real sample studies are conducted using the standard addition method, which exhibits accepted recovery. However, we believe that such methods are not reliable for real sample analysis. Thus, new methods should be developed.
- The depth mechanism for sensing applications should be studied.
- For SCs, stability is the major concern, which needs to be properly studied in depth.

We believe that future studies may involve the following key points for further research.

- Green and cost-effective methods need to be developed for scalability and practical applications.
- Electrochemical sensors can be combined with machine learning technology for the accurate analysis of environmental pollutants.
- Electrochemical sensors can be explored in smartphone-based devices for environmental monitoring.
- Flexible and wearable electrochemical sensors can be developed for environmental monitoring.
- MXene is a new material, and its potential is not fully explored. Thus, Bi_2_WO_6_ and MXene-based materials should be studied in depth, and a cost-effective and acid etching-free method should be developed for the transformation of MAX to MXene.

## Figures and Tables

**Table 1:** Performance of some previously reported electrochemical sensors.

Electrode Material	Analyte	L_OD_	Sensitivity	L_R_	Technique	References
Bi_2_WO_6_ nanoplates@SPE	BPA	20 nM	-	70 nM to 1300 nM	DPV	63
Bi_2_WO_6_ nanoplates@SPE	DES	15 nM	-	50 nM to 2100 nM	DPV	63
Nafion/HRP/3D-Bi_2_WO_6_/GCE	H_2_O_2_	180 nM	-	0.5 µM to 100 µM and 100 µM to 250 µM	AMP	64
Bi_2_WO_6_ NSs/GCE	Sudan I	5 nM	3.0563 µA µM^−1^	0.02 µM to 114.6 µM	DPV	65
TiO_2_/Bi_2_WO_6_/Ag/GCE	H_2_S	80 nM	-	0.5 μM to 300 μM	AMP (i-t)	66
Br-terminated 2D Bi_2_WO_6_ NSs	SMX	5.44 µM	-	-	DPV	67
Bi_2_WO_6_ (PVP + NaOH)/GCE	RF	12.151 nM	1.1415 µA µM^−1^ cm^−2^	0.03 µM to 457 µM	DPV	68
Bi_2_WO_6_	AA	77.85 mM	0.26 mM/mA	-	CV	69
MBLs/CS/Bi_2_WO_6_/MWCNTs-COOH/GCE	Ampicillin	0.17 nM	-	0.0007 µM to 0.05 µM and 0.1 µM to 10 µM	DPV	70
Bi_2_WO_6_	HQ	57 µM	-	200 µM to 5 mM	SWV	71
Bi_2_WO_6_	RS	4.3 µM	-	20 µM to 5 mM	SWV	71
Zn_3_(VO_4_)_2_/Bi_2_WO_6_	Cotinine	1 pM	-	1 nM to 5 µM	CV	72
Bi_2_WO_6_	Nicotine	13 nM	56.97 ± 2 μM^–1^	50 nM to 100 μM	I-V/Amp	61
CoTAPc-GO/Cu-Bi_2_WO_6_	DA	7.2 nM		0.05 µM to 5 µM and 5 µM to 250	PEC	74
ITO/Bi_2_WO_6_/CdS/Ab/BSA	Amyloid beta	0.068 pg mL^−1^	-	0.2 pg mL^−1^ to 50 ng mL^−1^	PEC	75
Bi_2_WO_6_@GNRs	HQ	7.51 nM	42.2 μA μM^−1^ cm^−2^	0.021 µM to 1550 µM	DPV	76
Bi_2_WO_6_@GNRs	CC	5.31 nM	54.14 μA μM^−1^ cm^−2^	0.021 µM to 1550 µM	DPV	76
BCN-200/rGO	4-NP	14 nM	12.86 μA μM^−1^ cm^−2^	0.2 μM to 100 μM	DPV	77
Bi_2_WO_6_/rGOS	4-NQO	0.006 µM	-	0.025 µM to 718 µM	Amp	78
Bi_2_WO_6_/Nb_4_C_3_Tx/ds-DNA/SPE	Pemetrexed	2.8 nM	-	0.01 µM to 100 µM	DPV	80
g-C_3_N_4_/Au/WO_3_	SARS-CoV-2 NP	3 fg mL^−1^	-	-	DPV	81
CoPc/Bi_2_WO_6_	Nitrite	0.063 µM	-	-	DPV/Amp	82
CoPc@Bi_2_WO_6_	Nitrite	0.12 µM	-	-	DPV/Amp	82
MIPs/Bi_2_WO_6_@GH/ITO	4-NP	5.78 × 10^−13^ M	-	5.0 × 10^−12^ M to 1.0 × 10^−7^ M	Amp	84

**Table 2:** Electrochemical performance of previously reported SCs based on Bi_2_WO_6_ and its hybrid composites as electrode material.

Electrode Material	Specific Capacitance	Electrolyte	Current Density	Cyclic Stability	References
3D-Bi_2_WO_6_/NF	1.12 F/cm^2^	2 M KOH	1.5 mA/cm^2^	10,000	88
BWS/RGO-12	1058 C/g	6 M KOH	3A/g	10,000	95
3D BiVO_4_/Bi_2_WO_6_	616.8 F/g	1 M KOH	1 A/g	4000	96
Bi_2_WO_6_(BWO)/ZnO/rGO	254.34 F/g	2 M KOH	2 A/g	-	97
BW/T@40 %	1153.3 F/g	2 M KOH	1 A/g	-	98
Bi_2_WO_6_-SnO_2_ quantum dots	812 F/g	3 M KOH	7 A/g	3000	99
Bi_2_WO_6_-NiO	398.2 C/g	3 M KOH	10 mV/s	2000	100
Bi_2_WO_6_ (BWO-2)	647 F/g	1 M KOH	5 mA/cm^2^	5000	89
Flower-like hierarchical Bi_2_WO_6_	255 F/g	1 M KOH	0.5 mA/cm^2^	850	90
Flake-like Bi_2_WO_6_	214 F/g	1 M KOH	0.5 mA/cm^2^	850	90
Spherical structure Bi_2_WO_6_	412 F/g	1 M KOH	0.5 mA/cm^2^	850	90
3D Bi_2_WO_6_/rGO	268.7 F/g	6 M KOH	0.75 A/g	1000	101
Bi_2_WO_6_/graphene	922 F/g	6 M KOH	3 A/g	2000	102
MIL-53(Al)/Bi_2_WO_6_	220 F/g	3 M KOH	0.5 A/g	5000	103
Bi_2_(WO_4_)_3_/aniline/CB	306 F/g	1 M KOH	1 A/g	-	104
Bi_2_WO_6_-g-C_3_N_4_	158 mF/g	6 M KOH	1 mA/cm^2^	1000	105
Bi_2_WO_6_/graphene	714 F/g	6 M KOH	4 A/g	-	106
MIL-53(Al)/Bi_2_WO_6_	554 F/g	6 M KOH	0.5 A/g	-	107
BWO-600	614 C/g	6 M KOH	2 A/g	10,000	91
CuBW80	1137 F/g	3 M KOH	0.5 A/g	10,000	108
Bi_2_O_3_/Bi_2_WO_6_	495.05 F/g	1 M KOH	1 A/g	2000	109
BW-BIC-PANI_200_	156 A/g	3 M KOH	1 A/g	4000	110
ABW@MXene	1420 F/g	1 M KOH	1 A/g	-	111
PVP-assisted Bi_2_WO_6_	462 F/g	1 M KOH	3 mA/cm^2^	500	93
HMT assisted Bi_2_WO_6_ NPs	708 F/g	1 M KOH	3 mA/cm^2^	5000	94

## Data Availability

No new data were generated. Authors are unable to provide data.
